# Teachers Responding to Pacific Community Voice: Supporting Relationships Through an Ecological Research Initiative

**DOI:** 10.1007/s40841-023-00285-4

**Published:** 2023-05-06

**Authors:** Cherie Chu-Fuluifaga, Martyn Reynolds

**Affiliations:** Te Herenga Waka, Victoria University of Wellington, Wellington, New Zealand

**Keywords:** Pacific education, Va, Pedagogy, Relational theory, Community voice

## Abstract

Pacific education is an area of priority in Aotearoa New Zealand. It involves the teaching of Pacific students by a workforce that is largely of European origin. Pacific communities value education and have the capability to contribute to the understandings of teachers as they seek to provide the kinds of service that communities want to see. This article reports on a Teaching and Learning Research Initiative (TLRI), *Learning From Each Other*. Leveraging talanoa as a dialogic research approach, the initiative examines the change value of Pacific voice in enhancing teacher understanding and promoting deliberate action to improve Pacific education. We present findings organised by spaces in which educators enact change using a contextualised revision of Bronfenbrenner’s ecological model as a mapping tool. What emerges is a sense of how non-Pacific educators’ growing Pacific-informed understandings support Pacific learners in personal, classroom and institutional spaces.

## Introduction

Pacific (or Pasifika) education, the education of students with links to one or more Pacific Island Nations (Airini et al., 2010) is a priority area for government in Aotearoa New Zealand, a response to sub-optimal experiences of Pacific students. Although Pacific peoples’ understandings of educational success have been sought (Alkema, 2014; Toumu'a, 2014), education remains largely mono-cultural (Gorinski & Fraser, 2006; Samu et al., 2019). Consequently, research that seeks equity by framing education aligned with Pacific thought and experience is valuable.

This article adopts a relational research approach to the education of Pacific students. Findings from a Teaching and Learning Research Initiative (TLRI) inquiry illustrate the potential of Pacific community voice to support teachers to re-think and change educational practice. The article opens with a positioning discussion that embraces the researchers, Pacific communities, parents and students, and teachers, and progresses to the conceptual underpinnings of the research. We then address the inquiry itself, paying attention to the research questions, approach, and ways of imagining the findings. Discussion is then framed through a series of socio-spatial locations that draw attention to the relationships involved. Finally, key insights and conclusions are given.

## The Research Context

### The Researchers

As researchers we value diverse experiences and learning. Cherie is an activist member of Pacific communities, is New Zealand-born of Tahitian and Chinese descent and based in tertiary education. She is also involved in Pacific education in the primary sector as a parent and educator. Martyn is Anglo-Welsh, a practitioner in education for over 35 years. Our partnership commenced when Cherie was Martyn’s doctoral supervisor. Together we have been learning to pursue our common goals—the enhancement of Pacific education and community autonomy. We focus this research in the relational spaces between researchers, Pacific communities, and teachers.

### Pacific Communities

The 2018 census reported 381,642 Pacific people living in Aotearoa New Zealand. However, in this context ‘Pacific’ is an umbrella term for diverse communities. Diversity takes many forms including ethnicity, birthplace, age, migratory generation, socio-economic status, and gender. Because Pacific students reflect this complexity, the term ‘Pacific education’, although perhaps helpful to administrators or as a heuristic for schools and researchers, can act to silence difference and mask diversity (Naepi et al., 2017; Samu, 2006) within and between Pacific communities. Here we use the term circumspectly in order to facilitate discussion.

Pacific students bring a wealth of knowledge and experiences from their communities and carry intergenerational aspirations, including for high quality education (Cunningham et al., 2022). However, their educational experiences are not optimal, affecting achievement (New Zealand Qualifications Authority, 2019), wellbeing (Office of the Children’s Commissioner, 2018), representation (Johansson, 2012; Siteine & Samu, 2011) and access to learning materials—exacerbated under COVID 19 restrictions (Mutch, 2021). Resources have been developed such as Tāpasa (Ministry of Education, 2018)—support for teachers of Pacific students, and the Action Plan for Pacific Education (Ministry of Education, 2020a). Developed in consultation with Pacific communities, the resources indicate the potential of Pacific people to guide the teachers of their children. This research suggests one way that teachers can be supported to respond deeply to matters which these resources raise.

### Teachers

The teaching force in Aotearoa New Zealand is largely of European origin (73%) and female (76%). Only 4% identify themselves as Pacific (Education Counts, 2021). Although the term European used for the majority group also masks diversity, it is clear that most Pacific students will not be consistently taught by Pacific teachers. Consequently, improvements in the education of Pacific students require changes in the practice of European teachers, and to a system of European origin that legitimises their everyday practice.

### The Research

In this research, all involved are knowledge holders and learners. The researchers know about previous studies and have experience of changemaking but seek pathways through which Pacific parent voice can change education. Pacific parents are willing and able to contribute to their children’s progress (Chu et al., 2013). We assume that Pacific parents know their own aspirations, cultural understandings and children but seek to support teachers to meet their children’s needs. We also assume that teachers know about what goes on in education and about many ways that learning happens but seek to transform their approaches for the benefit of Pacific students, parents, and communities.

Notwithstanding diversity, the relational focus of the research heuristically imagines Pacific education as a space where Pacific communities and largely European origin teachers can come into productive relationships based on the Pacific value of generosity (Rimoni et al., 2022). The research, therefore, seeks to creates a space where time, knowledge and commitment is warmly gifted by all in trustful ways to achieve the common goal of enhancing the education of Pacific students. This involves valuing learning, negotiation, and increasingly warm, close relationships.

Sustainable and effective change requires the transformation of hearts and minds so that teachers re-understand the education of their Pacific students in ways that are progressively closer to those of the Pacific parents and communities they serve. To do this, teachers need to be able to reflect on their practice and generate innovative ways of doing everyday things, leveraging their relationships with their schools’ Pacific communities as inspirational resources. Creating such opportunities is the core of this research.

## Conceptual Underpinnings

### Va

It makes sense for research that privileges Pacific voice to be structured using concepts that originate in the Pacific region. This research honours va, an understanding of relational space at home in various forms in Samoa (Wendt, 1999), Tonga (as vā) (Koloto, 2017) and elsewhere. Va points to the self as social/relational rather than individual (Mila-Schaaf, 2006; Vaai & Nabobo-Baba, 2017) and to interconnections between spiritual, social, and physical dimensions of relational space (Anae, 2016).

The ethics of understanding relationships as relational space are indicated by the Tongan reference tauhi vā (Koloto, 2017) and the Samoan, teu le va (Airini, et al., 2010; Anae, 2010a). Teu le va has been rendered in English as keeping relational spaces tidy (Anae, 2010b), well-configured, and nurtured (Airini et al., 2010). The stated aim of the research is to teu le va by facilitating and investigating the support necessary for teachers to improve the education of Pacific students in their context in line with the aspirations of local Pacific people(s).

### Space

Because the research is underpinned by va, space is not empty but simmering with relationships, ethical responsibilities and opportunities. Wendt (1999) says ‘va is the space between, the betweenness, not empty space, not space that separates but space that relates, that holds separate entities and things together in the Unity-that-is-All, the space that is context, giving meaning’ (p. 402). Mila-Schaaf (2006) writes of va as space we feel through imagination rather than see. Tuagalu (2023) discusses va as fields in which forces provide connection. In his account, such forces include mana (divine energies), tapu (sacredness) and alofa (love). This research seeks to facilitate positive and ever-closer connections between Pacific communities and teachers on multiple levels. We trust teachers’ learning from Pacific community voice to challenge existing practice and re-populate classroom and school spaces with actions that embody care, respect, and love.

### Community

The research is underpinned by a conceptualisation of community that is multi-layered and contextually fluid, embracing both separation and connection to mirror the dynamics of the va. There is relevance in the idea of Pacific communities being distinct from the teaching community; a key aim of the research is to teu le va between these groups. However, other connecting layers of community exist in Pacific education. These include the school community to which Pacific parents, students and teachers all belong in various ways.

The concept of relational positionality (Fasavalu & Reynolds, 2019) captures the ways that people can come closer together as members of a shared community through interaction, learning and enhanced mutual understanding. It is the aim of the research to support increased relational closeness in school communities.

## The Inquiry

### The Study

This account of *Learning From Each Other* is the research story of a group of eight teachers engaged with their schools’ Pacific communities. The teachers were drawn from one faith-based Kahui Ako (group of schools across primary and secondary sectors) (KA) in urban South Island | Te Wai Pounamu, Aotearoa New Zealand. They volunteered to participate as teachers-as-learners in the research.

Two research questions (RQs) the initiative set out to answer were:

RQ1: What roles can Pacific concepts and voices (communities’, families’, and students’), and practitioners’ cultural humility play in developing Pacific education as a productive partnership?

And

RQ2: What benefits can be gained for Pacific students from enhanced relationships between teachers’ Pacific conceptual and lifeway knowledges and their developing practice?

RQ1 values partnership as power sharing and recognises that while Pacific origin information (voice and concepts) has the potential to care for the va of Pacific education, these gifts must be received. Cultural humility (Foronda et al., 2016) is centred on openness to learn. Thus, being a teacher-as-learner is an element of teachers’ self-development and service to others.

RQ2 pays attention to the benefits to Pacific students from the learning of teachers-as-learners. The research aim of caring for the va of Pacific education assumes that learning from Pacific people(s) will facilitate helpful change in teachers’ practice.

The research began by the host school asking their Pacific communities about the parameters of consultation through ‘cultural brokers’, Pacific people already known to them. Consequently, a KA community research fono (consultative meeting) took place. Around 100 Pacific students, families and community members joined about 12 teachers and the two researchers to participate in a sharing of ideas for one evening. The programme included performance by Pacific students, speeches of welcome and response, a shared meal, and opportunities to talk.

### Talanoa

*Leaning From Each Other* is dialogic research informed by talanoa. In Pacific communities, talanoa is a form of conversational interaction that pays attention to the ethics of the va (Havea et al., 2021). Vaioleti (2013) describes talanoa in research as an oral form that moderates power relations. It involves the empathetic alignment of the emotional and spiritual states of researchers and participants in a space made safe for all (Farrelly & Nabobo-Baba, 2014). In this research we use talanoa to denote our overall dialogic approach to shaping and conducting research with authentic purpose.

We frame two layers of dialogue as talanoa because of the relational focus and deliberate construction of safe spaces in each. First, talanoa took place among Pacific parents and community members at the community research fono. Second, small group PLD spaces were progressively shaped by the researchers as talanoa in the form of safe critical conversation among teachers. In these spaces we sought an atmosphere of friendship free of judgement. As newcomers to talanoa, teachers were offered experiential learning in Pacific practice.

A valuable dialogic relationship existed between the two levels of talanoa. The community research talanoa was supported by optional prompts designed to support future Pacific community-teacher dialogue. These included ‘What advice do Pacific parents want to offer their children’s teachers?’ A scribe recorded notes or verbatim comments during the 40–50 min session so that a rich information set became available to support a mediated dialogue (Nakhid, 2003; Reynolds, 2017) facilitated by the researchers between Pacific families and teachers. Mediated dialogue acknowledges issues of power by creating opportunities for asynchronous communication. This involves shifting the words and/or ideas of one group derived from an initial engagement to another group in a different, but connected, time and space. The result of the second engagement is subsequently fed back to the originators in a dialogic loop.

In *Learning From Each Other,* the second stage of the mediated dialogue involved talanoa among teachers (PLD talanoa) centred on voice from the community research talanoa. Over one year and into a second, researchers and teachers met for talanoa nine times. In the first year, each two hour PLD talanoa was an exercise in sense-making of Pacific parent voice by teachers supported by the researchers and on occasion, an ‘outside’ Pacific person. These PLD sessions were organised according to themes that emerged from the community research talanoa: Pacific identity; school culture; student–teacher relationships; parent-teacher relationships; and success and cultural norms*.*

A typical PLD talanoa from the first year involved a recap through which teachers reflected on their previous learning, the presentation by the researchers of themed information from the community research talanoa, dialogue about the significance of what parents and community members had said, and opportunities to reflect on new learning and ways teachers might respond. In the second year, the teachers invited members of their local Pacific communities to contribute during three whole day PLD experiences to deepen their understanding of the information from the community research talanoa.

Comments from the community research talanoa confirmed the value of mediated dialogue. Throughout the evening, Pacific speakers described how respect, discomfort and percieved power in educational spaces can combine to create distance between themselves and teachers so that *parents and children may be too shy to approach teachers.* Conversely, comments such as *thank you for using your ears* and *this (fono) is a wonderful idea* suggest that the community research talanoa provided Pacific parents with more comfortable opportunities to talk. In response to the PLD, teachers made comments such as* I immediately think this is being a really safe space… and I think when we have stupid questions…that it’s okay to not shame us when we ask,* suggesting the value of talanoa within a mediated dialogue to them.

The final COVID 19-delayed aspect of the dialogic loop involved a service of blessing, teachers describing their learning and some of their actions, questions, thanks and food, attended by around 60 members of the KA community, including members of local Pacific communities.

## Imagining the Findings

This article draws on information from PLD talanoa. The information from PLD talanoa was iteratively coded following Informed Grounded Theory (IGT) (Thornberg, 2012) by testing the validity of spatial sensitising concepts such as school and classroom, and temporal aspects such as school rituals. Due to scale and ethics, individual speakers are not identified and some redaction has occured. What emerges are space-focussed sets of teachers’ deliberate actions. These are accompanied by explanatory reflections. To imagine the potential of the information in relational terms, we turn to Christensen’s (2010) enhancement of Bronfenbrenner’s (Shelton, 2018) ecologic approach as a basis for a model to plot the effect of Pacific voice on teacher actions through PLD in this initiative.

### Ecologic Modes of Relational Influence

Bronfenbrenner’s interest in ecological systems focused on child development. The child is at the centre of rings of influence that represent social distance. A micro-system of immediate points of contact, for example, family, school and church, has the most direct influence on the child’s development. Outside this is the mesosystem, a space permeated by relationships between the micro- and exosystem. The exosystem accommodates factors that affect a child indirectly, such the education system. The macrosystem involves culture(s) and ideologies that permeate the various layers of the model. The chronosystem takes account of changes and influences over time. From Bronfenbrenner’s model this research values individual development as a relational matter; relative distance as a way of imagining degrees of influence; the integrated nature of influence notwithstanding distance; and attention to time to describe the dynamic status of systems and their relations.

Christensen (2010) proposed a modification to Bronfenbrenner’s model for the professional development of adults by adding an intra-level to represent attributes such as resilience. Christensen also introduced networks, represented by web-like lines and nodes to map influential relationships. This research values the intra-level to account for ideological postures teachers bring to PLD including the desire to learn and resilience in the face of the educational status quo. Networks helpfully draw attention to the way influential relationships straddle system boundaries.

**Fig. 1:**
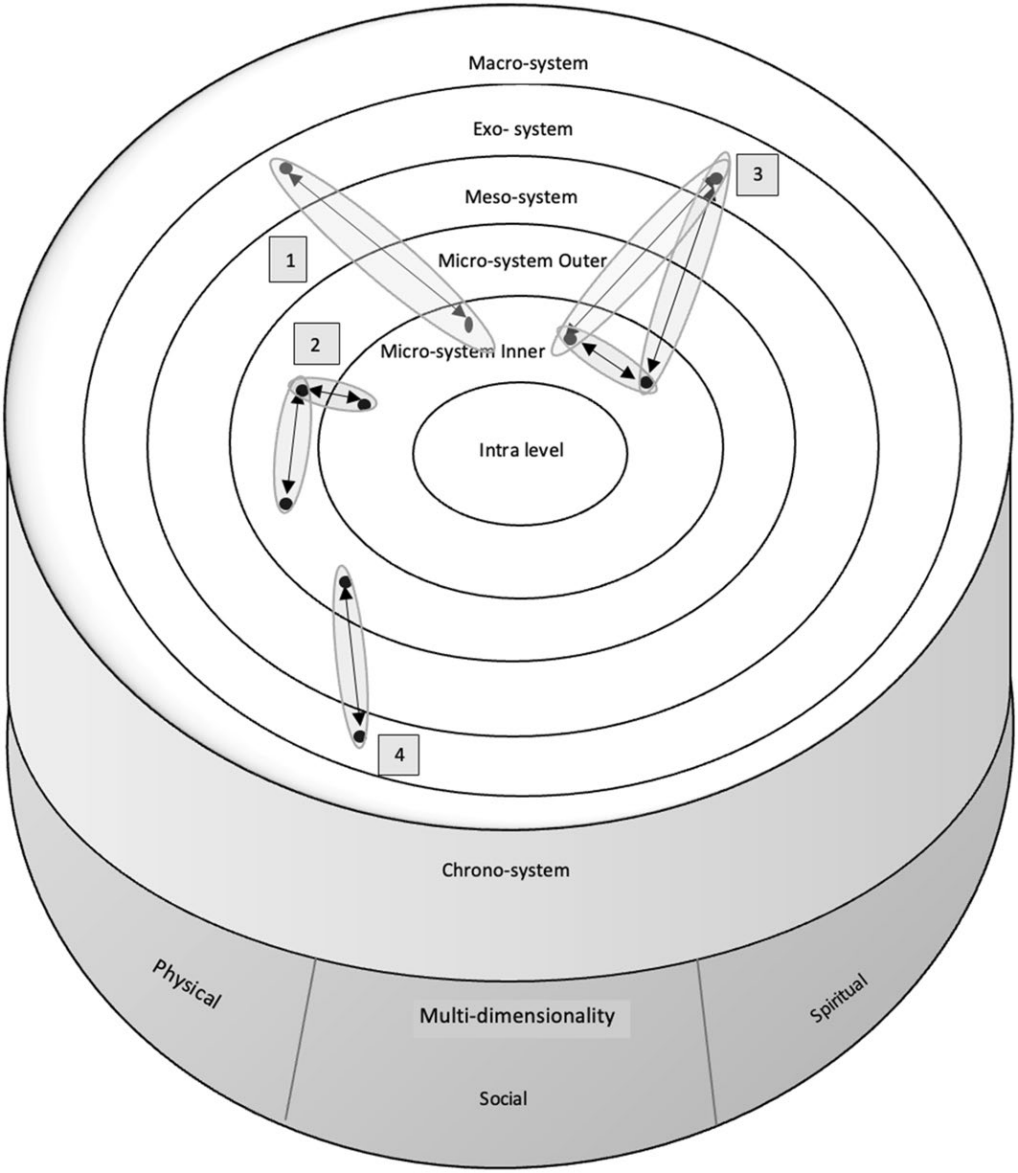
Imagining the va through the lens of Bronfenbrenner’s and Christensen’s Ecological Model

We propose further adaptions to Christensen’s modification, changing networks to va represented by spaces. In Fig. 1, double-headed arrows in va spaces represent the dynamics of connection and separation. By this means we map the potential of learning, including that facilitated by PLD, to enhance closeness in relational connection. This draws attention to well-configured connective relational spaces as the key to successful to education in Pacific understandings and to teu le va as an ethic (Anae, 2019) in education.

We adjusted Bronfenbrenner's concentric circles to address the specific spaces that emerged from coding the PLD talanoa. We describe the classroom (inner-micro) and school (outer-micro) within the micro-system; spaces where teachers have direct influence. The mesosystem represents the porous nature of school community within our conceptualisation since schools can work to make their communities more inclusive. The exosystem deals with links between the teacher’s immediate school/classroom context and other enviroments in which they have less active influence, such as Pacific communities. The macrosystem includes cultural elements such as the logic of the educational status quo. The chronosystem accounts for intergenerational issues in the education of Pacific students and future positive developments. Finally, we represent the multi-dimensional nature of va through a hemisphere that depicts the holistic connections between the physical, social and spiritual. The numbered examples within the model will be discussed during the findings section.

## Findings and Discussion

In this section, stories drawn from PLD talanoa are counterpointed by Pacific voice from the research talanoa to draw attention to its potential to affect teachers’ thought and practice. We begin with the inner-microsystem of the classroom va.

### The Inner-Microsystem: The Va Between Pacific Students and Teachers

#### Classroom Materials

A key feature of the research talanoa was a call from Pacific parents to teachers to make classrooms places where Pacific children feel comfortable. This was expressed in several ways including:

- Allow children to have a greater sense of belonging
- We value being strong in who you are and where you come from, and
- Identity and self-belief are important.

A significant and frequent way teachers describe responding to these notions involved altering classrooms materials. Three varied examples illustrate the scope.

First, teachers report deliberately incorporating elements of Pacific home life to shape the way Pacific students experience learning. For example,

I did some little bit of investigating of some names of relatives, in our maths we sort of put [name], ‘Oh that’s my grandma’, and ‘Oh that’s my little brother’, and that just wasn’t anything much different, [I] just made a conscious effort of using all of the children’s names, not just the Pasifika kids… they became more invested, it was just like an extra 10% of that without extra work.

Time spent gathering and using family names has, according to this teacher, produced enhanced engagement. Perhaps this is because the mathematics became less abstract when it was located in familiar relationships where children belong. The students’ reactions seem to indicate the significance of hearing of a relative in school even in a fictional context. This echoes the significance of contexts such as family and cultural values as described by Hill et al. (2019). The purposeful action of the teacher acknowledges that the education of Pacific students is a collective affair with students relationally entangled in larger units (Matapo, 2017). This is represented as #1 in Fig. 1—the teacher has drawn attention to family relationships so that they become salient in the classroom between the student and the teacher in learning.

Teachers also describe making time and space for Pacific students to bring their skills and interests from home to school. An example from the primary sector involves music:

And [the student] wanted to show his drumming, so he got up, and he’s got a bit of a difficulty with speech, but I’ve never seen him so articulate, such great diction, such great clarity as when he was explaining how the drums worked, and when you push on a different beat, and the kids were just fizzing…

This suggests the significance of reciprocity in providing opportunity and encouragement for Pacific students to introduce activities from their own milieu into the classroom culture. For this child, the consequence seems to have been that a strength and element of identity support an area of relative weakness in a transformative way.

A final example is a teacher deliberately shaping the classroom as a space that invites home based knowledge and experience:

I prioritised… one morning a week of art, storytelling, and drama at the beginning of the day. And some of the richness of what we did actually we could use for writing… it was so, belonged to them that it was easy to use… there was [an] exploding, and shattering, and some of this great vocab that came out. It was like, ‘The challenge is to write a story tomorrow with some of these words in it’.

Prioritising arts as a way of bringing students’ experiences and self-expression into classrooms may seem habitual for many teachers. However, this deliberate action took place in an environment where the pressure from the macrosystem of new ways of teaching reading and mathematics assert alternative, highly structured priorities. In this case, the parental voice from the research talanoa supported the teacher to make space for ‘*where you come from’* in arts and literacy work so that other priorities were held in check. The story illustrates the way that priorities sometime conflict in the classroom—attending to Pacific identity, language, and culture, and using new methods in literacy are all educational priorities.

Other examples of changes in materials described by teachers included incorporating Pacific languages into spelling and reward protocols, purposeful attention to the correct pronunciation of Pacific learners’ names, and sourcing Pacific materials for reading and display.

#### Pedagogy

Pacific parents also discussed pedagogy as relational activity in the research talanoa:

- Ask the children lots. Do it one-on-one. Ask what they are struggling with
- We value a teacher who believes in them, who takes time to really understand them
- Take notice that students have a voice that they may be scared to show
- Don’t always give us the easy options. Challenge us and our learning. Aim high.

A frequent aspect of successful engagement in the Pacific education literature is reducing relational and physical distance through individual and/or small group teaching (Fletcher et al., 2011; Reynolds, 2017). In this narrative, a teacher describes the effect on one student of valuing closeness:

So, one of my things was… getting alongside our students. So, one child in particular is quite quiet and I never really heard his voice, his strong voice. Getting alongside him is really effective. So, I've been doing it a lot, and he's become a bit more brave in class to give me his opinion and his thoughts. So that's been really good.

Increased bravery suggests that the strength of the teacher-student relationship has enabled the child to flourish in a range of times and spaces. Individual attention has fostered a journey to independence.

A second deliberate pedagogical response to Pacific parent voice focusses on communicating high expectations.

We were giving speeches this week and last week and… a Pacific student was saying ‘I don’t want to get up there and do this…’. And I said, ‘I think you’re more than capable of doing this, I know you don’t feel actually comfortable doing it, but I’ll be here right beside you or you can take a friend with you, you’ve written this incredible speech, you’ve got this!’

In this case, an issue for the student is matched by a collective solution from the teacher. Expectations are maintained by offering changes that make success more comfortable and therefore more likely. This is represented as #2 in the model—the teacher has made an asset of collectivity in the form of peer relationships that exist in the school so that peer relationships become salient as an aspect of the work in the classroom.

Taken together, these extracts from the PLD talanoa paint a picture of teachers responding in context to Pacific parent voice so that the classroom benefits of participation, engagement, personal development and expression of voice accrue to Pacific (and other) students.

### The Outer-Microsystem: The Va Between Pacific Parents and the School

The research talanoa revealed Pacific parents’ desires for more effective partnerships with teachers based on enhanced communication. Comments from the community research talanoa include:

- Authority in the form of teachers/principal can be scary. Think of friendly spaces
- Create that bond between teachers and parents for communication, so that everyone is on the same page, and
- It’s important that we feel OK to see the teacher and talk about a child struggling.

Three examples illustrate the way teachers deliberately rethought school protocols to draw parents closer to the school. The first involves the way a teacher re-approached a potentially difficult conversation about a Pacific child’s learning to address the habitual power balance of such encounters:

I think if I had that conversation behind the chair, it would’ve been quite cold. Whereas because we get to sit side by side on the little kids’ couch… we were really close, we were kind of knee to knee, she had her mask on and… it felt like I was talking to a friend - or it changed the relationship I feel. And then as things have come up, it’s opened up that conversation… I can actually flick her a wee email and tell her about things that have happened. Whereas before…I think it would’ve been very defensive back. Whereas now it’s like, ‘Oh yes, we’ve noticed that happening too, have you tried this? Because this works at home when he does that.’

This account suggest how physical space reconfigured in pursuit of partnership can shape social relationships. This is represented as #3 in the model—the result of improved communication channels is information from the parent to help the teacher structure how they behave in the classroom with the aim of relating more closely to the student.

In the second example, a teacher discusses how they altered home-school communication channels in two ways:

Another thing that I’ve been trying to do is, with parents… communicating more rather than just relying on sending a note home or via the child… to actually ring up… [And] a couple of my children and parents do come into school so I’ve been trying to make a point of actually talking to them, rather than just relying on sending messages home…I find out more information that way, and it’s been really good...

First, the teacher seeks more direct connection. The telephone is a more personal interaction than sending messages. Second, the teacher recognises Pacific parents in the school as an opportunity to interact to be capitalised on. The teacher reports these reshaped encounters led to useful learning.

In the third example, a teacher reports revised ideas of responsibility around formal reporting.

I think we're starting to shift about the way we communicate progress to parents, even though we're still very traditional in the way that we do it but we're actually making a point of it, instead of saying ‘Oh well they haven't read it but we're not going to worry about it’ - we looked up and we've seen who hasn't [read the report] … We just don't think ‘Oh they're not interested in the education of their child’. It's just we have to think of different ways.

Here, while the form of communication has not altered, the relationship between the school and parents has changed in two ways. First, an assumption that absolved the school of responsibility for communication has been disturbed, undercutting the stereotypical macrosystem notion that Pacific parents are not concerned about their children’s education (Hemi et al., 2021). Second, the school is taking responsibility for knowing who has accessed reports and for finding alternative avenues of communication where needed. The school has become the site of critical attention rather than the behaviour of Pacific parents. When those with the power to change school communication accept responsibility for the outcomes of their choices, partnership becomes more likely.

In these examples, schools have given up aspects of power regarding what consultation should look like in interviews, the place of personal interaction in messaging, and how reporting is evaluated. These changes aim to support the kinds of partnership described by parents at the research talanoa.

### The Exosystem: The Va Between Pacific Communities and Education

As might be expected, the results of research aimed at enhancing relationships between teachers and Pacific parents focuses attention on relationships between Pacific students, their parents, and teachers. However, Pacific parents are well aware of wider routes to shape partnership. Suggestions from the research talanoa included:

- Pacific parents need to be on the BOT [Board of Trustees] and need to be involved in more than a token way, and
- Get different people involved from community.

In this account, a teacher who is also a school leader explains how the school board has modified its practice to be more inclusive of Pacific (and other) representation.

We had… a strategic board meeting last night and all our different cultural groups introduced themselves with pride in who they were. They introduced and they said, one said their name and that ‘I’m a Cook Islander’…. So, there’s those things that, because we’re talking about it [Pacific involvement in education] all the time that’s showing that it’s valued, and we’ve got that idea and it’s made me think in terms of our… cultural community strategic goal. We had that strategic meeting on what that’ll look like next year. So, that’s been my learning…

As the host of the research, this school’s leader was significantly involved with the cultural brokers. From these interactions, the school leader gained a model of engagement based on talanoa. It seems they have recognised the value of safe spaces in modified meeting practice. As a consequence, in the meeting described relationships and presence were valued through opportunities for those involved to participate in their own way. The active participation of Pacific people in board meetings is essential if Pacific community voice is to re-shape education at the institutional level.

Confidence was a factor in the changes from the first to the second year of the research. In the second year, the teachers took responsibility for involving local Pacific community members. These included Pacific students, teachers, and an artist; and staff and children at a Pacific Early Childhood Education (ECE) centre. A visit to see Pacific ECE teachers working in a Pacific-centred environment was an opportunity to better understand where many of the KA’s students had and would come from. Community level involvement of this nature provides teachers with experiential learning, supports relationships of value in the future, and illustrates the potential of prolonged talanoa as a learning process to develop teachers’ confidence to access local opportunities to learn from Pacific people. Community involvement is represented by #4 on Fig. 1—the teachers learn by extending their reach into Pacific community educational provision.

### Intra-system: The Va Between Understanding and Action

As described above, the stories from the PLD talanoa offer a window on changes that show the potential of Pacific parent voice to help teachers re-think and devise new practice. Attention to the intra-system, the va between understanding and action, exposes a number of personal growth points for teachers—sites where re-understanding has made changes in approach logical. Four nodes of understanding are presented here to illustrate the dynamics involved: deliberateness, scale, relational thinking, and cultural safety. These go some way towards answering RQ1 because they suggest how productive partnerships can result from Pacific concepts and voices interacting with teachers’ cultural humility or willingness to learn from others.

### Deliberateness

I think in the teaching and the learning, it’s being aware of how I’m doing things and what I say, that just it’s developed that awareness, I think.

Being deliberate can turn a teacher’s good practice into a consistent experience for Pacific students. That is, while many effective practices may be present in classrooms, attending to the way Pacific parents describe their aspirations can encourage teachers to use these in more frequent, organised, self-monitored and regular ways. These qualities shift occasional or sporadic actions into the core culture of the classroom.

### The Significance of the Small Scale

It’s thinking about those culturally responsive practices and those little, tiny things that I’ve changed that I feel like hasn’t been much of a change, has been massive for some of my parents…

Changing small scale everyday practices can produce powerful, sustainable improvement. An effect of the macrosystem visible at times in the PLD talanoa was teachers’ anxiousness that they had *not understood* or not got the *important pieces* because they were not developing a new *silver bullet* for Pacific education in their schools. This anxiety seemed to be fuelled at times by pressure from leaders in their schools for a simple, comprehensive ‘problem-solved’ outcome from the research (Edwards & Krishnan, 2016). This implies that the education of Pacific students is understood as a problem to be solved by ‘targeting’ Pacific students with a simple or one-size-fits-all solution. What is needed is for education to learn incrementally and continually from the communities it intends to serve.

Literature points to the value of small-scale changes in classroom practice (Reynolds, 2017; Siope, 2011) for Pacific students. Small changes are powerful precisely they are sustainable, every day, and consequently influential. Change that may seem small to a teacher may be of great significance to members of Pacific communities. Examples include signalling changed power dynamics through new seating arrangements at interviews; and signalling the value of the individual to the teacher through more frequent one-to-one interaction. Each example is a small act to teu le va in significant ways.

#### Relationships

So, the first one is understanding, and it’s understanding place and time, and space of the people that I’m with… And then co-instruction - what are we putting into this between us? What are the gifts being offered? What am I taking? Who’s taking, giving more?

Understanding education as gift giving in a relational space draws attention to reciprocation and partnership. Much has been written about the ‘factory system’ of education in which knowledge is transmitted and reciprocation is not valued. When Pacific students are seen to carry gifts (Tuagalu, 2008) and teachers accept these through ako (Ministry of Education, 2007) or reciprocal teaching and learning, education becomes much more of a partnership. This in turn validates Pacific students and their experiences.

#### Cultural Safety and Care

I immediately think, ‘Is this being a really safe space?’ … and I think when we have stupid questions [in the talanoa] that it’s okay… I think that will create allyship, and understanding, which will benefit the children.

Recognising the value of talanoa as a PLD process can provide experiential learning that exposes issues of cultural care and spiritual safety to teachers who are normally safe in their educational spaces. If teachers realise their feelings regarding safety are also applicable in classroom learning environments, this can help them become allies to Pacific students. Allyship as partnership suggests being on ‘the same side’ of learning through shared goals. This requires an appreciation that despite intent, feelings of insecurity can make education feel like a divided event where one party is safer than the other. Embodied learning about safety from talanoa as a process can help teachers deliberately avoid assumptions or stereotypes from the macrosystem about Pacific students’ academic ability, stereotypes clearly expressed as concerns by Pacific parents in the research talanoa.

Taken together, these growth points show the significance of confidence, incipient understanding of Pacific concepts (such as va and talanoa), and time for change to embed. These features can lead to deep and potentially lasting changes in how teachers understand and enact their roles, relationships and professional activities in regard to Pacific students, parents and communities.

### Mesosystem: The Ecology of the Pacific Education Va—Key Insights

If Pacific education is imagined as a va between Pacific students, parents and communities and largely European teachers, holism is a key concept to appreciate. Consequently, we now turn to the mesosystem as a way of approaching RQ2 and accounting for the benefits to Pacific students from enhanced relationships between teachers’ growing Pacific conceptual and lifeway knowledges and their developing practice.

Pacific educational research needs to make a difference in students’ educational experiences in order to be valid (Visser et al., 2007). Changes made within the inner-microsystem have a direct effect on Pacific students. However, an ecological approach to Pacific education stresses interconnectedness, and places value on indirect influence. Some changes take time and/or create further ripples before benefits become visible. The failure of education to deliver for Pacific people is a deeply engrained, indirect, intergenerational issue.

Teachers’ stories suggest that Pacific parent and community voice has supported them to partially de-privatise the classroom and school so that education has moved closer to wider Pacific community(ies). These changes foster a situation where mutual learning becomes increasingly possible, fueling the kind of trust-based powersharing that converts closeness into reciprocation and supports mutually appreciative partnerships.

Enhanced communication between home and school, and the active involvement of Pacific people(s) in partnership-like relationships in education have the potential to support the better alignment of Pacific students’ experiences of school and home. Where Pacific ideas and aspirations become central in education, belonging for Pacific students in school can be enhanced with profound consequences for engagement, participation, and learning (Ministry of Education, 2020b).

The mesosystem captures the kind of cultural change that makes deep shifts possible when changes in other systems are aligned. For instance, school logic can shift from deficit to strength-based approaches; diversity can be valued as a daily source of education rather than as an occasional exotic interest; and the ‘way we do things’ can become habitually open to interrogation based on outcomes rather than intent. Changes of this order are those required to re-think Pacific education moving forward. Steps towards power sharing partnerships are valuable drivers of change.

## Conclusion

At the core of this article is the potential of Pacific parent voice to support teachers to use their professional skills and experiences to re-align education in ways that honour Pacific perspectives and aspirations. An account has been offered from *Learning From Each Other,* a small scale TLRI designed to draw on Pacific wisdom in the form of talanoa, va and voice.

Pacific education has been imagined as a va between Pacific parents, students, and community members, and teachers so that teachers’ enacted learning operates to teu le va—to care for the relational space of Pacific education. Although limited in size, the research suggests that under appropriate circumstances, benefits for all can accrue from talanoa between those invested in the education of Pacific students.

The research points to the value of talanoa in several ways. Talanoa is an appropriate and therefore effective way of consulting Pacific community members as a form of engagement with schools, validating Pacific peoples’ belonging in the wider school community. Talanoa with community also makes local contextual information available to teachers, providing a pathway that supports teachers to embrace aspects of education highlighted by Pacific communities as important in Tapasā (Ministry of Education, 2018). For example, confidence developed through the talanoa process can produced teacher behaviour that ‘Demonstrates a strengths-based practice, and builds on the cultural and linguistic capital Pacific learners, their parents, families and communities bring’ (Turu 1.10, p. 11); and ‘Engages with Pacific learners, their parents, families and communities in all aspects of teaching and learning pathways' (Turu 2.10, p. 13). Experience of talanoa and learning about va encourages thinking that ‘Uses… different Pacific conceptual models and frameworks as a reference and guide for planning, [and] teaching' (Turu 3.11, p. 15). Talanoa also leads to warm relationships that can cross boundaries and brings potential for long term collaboration between the Pacific and teaching communities as members of wider school communities focused on constructing education of benefit to all those involved.

A productive relationship between Western-origin ecological theory and va has enabled actions and benefits to be plotted in educational and relational spaces, reflecting the partnership that is Pacific education. Classroom belonging, excitement and engagement; power sharing; mutual learning; and opportunities to extend representation have been described in a context where the ever-present macrosystem of competing priorities, stereotypes and inertia are powerful. What remains for future research are issues of scale—how to create similar opportunities across more KA, and depth—how to support re-thinking that is more focussed on the level of systems as well as the individuals that operate them. Despite (and because of) these issues, future research that honours the skills and knowledge of all involved so that learning from each other drives sustainable change has much potential to create progress on the Pacific education journey.
